# Elucidating the Role of Lipids in the Aggregation of Amyloidogenic Proteins

**DOI:** 10.1021/acs.accounts.3c00386

**Published:** 2023-10-12

**Authors:** Dmitry Kurouski

**Affiliations:** † Department of Biochemistry and Biophysics, Texas A&M University, College Station, Texas 77843, United States; ‡ Department of Biomedical Engineering, Texas A&M University, College Station, Texas 77843, United States

## Abstract

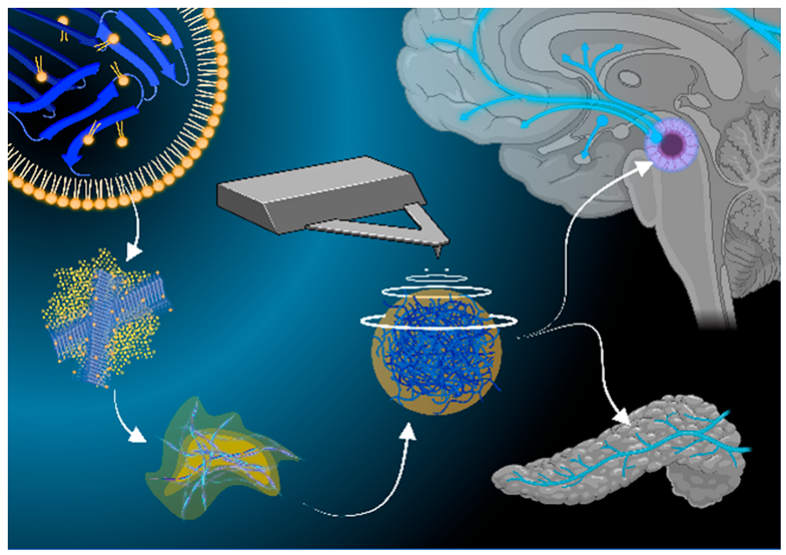

The abrupt aggregation of misfolded proteins is linked to the onset and spread of amyloidogenic diseases, including diabetes type 2, systemic amyloidosis, and Alzheimer’s (AD) and Parkinson’s diseases (PD). Although the exact cause of these pathological processes is unknown, a growing body of evidence suggests that amyloid diseases are triggered by misfolded or unfolded proteins, forming highly toxic oligomers. These transient species exhibit high structural and morphological heterogeneity. Protein oligomers can also propagate into β-sheet-rich filaments that braid and coil with other filaments to form amyloid fibrils and supramolecular structures with both flat and twisted morphologies. Microscopic examination of protein deposits formed in the brains of both AD and PD patients revealed the presence of fragments of lipid membranes. Furthermore, nanoscale infrared analysis of *ex vivo* extracted fibrils revealed the presence of lipids in their structure (Zhaliazka, K.; Kurouski, D. *Protein Sci.***2023**, *32*, e4598). These findings demonstrated that lipid bilayers could play an important role in the aggregation of misfolded proteins.

Experimental findings summarized in this Account show that (i) lipids uniquely change the aggregation rate of amyloidogenic proteins. In this case, the observed changes in the rates directly depend on the net charge of the lipid and the length and saturation of lipid fatty acids (FAs). For instance, zwitterionic phosphatidylcholine (PC) with 14:0 FAs inhibited the aggregation of insulin, lysozyme, and α-synuclein (α-Syn), whereas anionic phosphatidylserine with the same FAs dramatically accelerated the aggregation rate of these proteins (Dou, T., et al. *J. Phys. Chem. Lett.***2021**, *12*, 4407. Matveyenka, M., et al. *FASEB J.***2022**, *36*, e22543. Rizevsky, S., et al. *J. Phys. Chem. Lett.***2022**, *13*, 2467). Furthermore, (ii) lipids uniquely alter the secondary structure and morphology of protein oligomers and fibrils formed in their presence. Utilization of nano-infrared spectroscopy revealed that such aggregates, as well as *ex vivo* extracted fibrils, possessed lipids in their structure. These findings are significant because (iii) lipids uniquely alter the toxicity of amyloid oligomers and fibrils formed in their presence. Specifically, PC lowered the toxicity of insulin and lysozyme oligomers, whereas α-Syn oligomers formed in the presence of this phospholipid were found to be significantly more toxic to rat dopaminergic cells compared to α-Syn oligomers grown in the lipid-free environment. Thus, the toxicity of protein oligomers and fibrils is directly determined by the chemical structure of the lipid and the secondary structure of amyloidogenic proteins (Dou, T., et al. *J. Phys. Chem. Lett.***2021**, *12*, 4407. Matveyenka, M., et al. *FASEB J.***2022**, *36*, e22543. Rizevsky, S., et al. *J. Phys. Chem. Lett.***2022**, *13*, 2467). Experimental results discussed in this Account also suggest that amyloidogenic diseases could be caused by pathological changes in the lipid composition of both plasma and organelle membranes, which, in turn, may trigger protein aggregation that results in the formation of highly toxic oligomers and fibrils. Finally, the Account discusses the effects of polyunsaturated FAs on the aggregation properties of amyloidogenic proteins. Experimental findings reported by the author’s laboratory revealed that polyunsaturated FAs drastically accelerated the aggregation rate of both insulin and α-Syn as well as strongly changed the secondary structure of amyloid fibrils formed in their presence.

## Key References

- ZhaliazkaK.; KurouskiD.Nanoscale Imaging of Individual Amyloid Aggregates Extracted from Brains of Alzheimer and Parkinson Patients Reveals Presence of Lipids in Alpha-Synuclein but Not in Amyloid Beta(1–42) Fibrils. Protein Sci.2023, 32, e459810.1002/pro.459836823759 PMC10019452.^1^ Nanoscale analysis revealed the presence of lipids in the *ex vivo* amyloid fibrils.
- DouT.; ZhouL.; KurouskiD.Unravelling the Structural Organization of Individual Alpha-Synuclein Oligomers Grown in the Presence of Phospholipids. J. Phys. Chem. Lett.2021, 12, 4407–441410.1021/acs.jpclett.1c0082033945282 .^2^ Lipid bilayers uniquely altered the secondary structure of insulin fibrils that were formed in their presence.
- MatveyenkaM.; ZhaliazkaK.; RizevskyS.; KurouskiD.Lipids Uniquely Alter Secondary Structure and Toxicity of Lysozyme Aggregates. FASEB J.2022, 36, e2254310.1096/fj.202200841R36094052 PMC10427241.^3^ This work demonstrated that phospho- and sphingolipid bilayers could change the aggregation rate of lysozyme as well as alter the secondary structure and toxicity of lysozyme fibrils.
- RizevskyS.; MatveyenkaM.; KurouskiD.Nanoscale Structural Analysis of a Lipid-Driven Aggregation of Insulin. J. Phys. Chem. Lett.2022, 13, 2467–247310.1021/acs.jpclett.1c0401235266717 PMC9169669.^4^ This work showed that phosphatidylserine and phosphatidylcholine altered the secondary structure of α-synuclein oligomers.

## Introduction

Protein deposits are hallmarks of numerous amyloid diseases, including Alzheimer’s (AD) and Parkinson’s (PD) diseases. AD is one of the fastest-growing neurodegenerative diseases, projected to strike 14 million people by 2060 in the U.S. alone.^5^ In 2022, over 6 million Americans age 65 and older were diagnosed with this pathology, with estimated costs that are upwards of $320 billion, making effective neuroprotective treatments an urgent and unmet need.^6^ In 2023, AD-related costs exceeded $345 billion per annum in the U.S. alone, with the projected costs exceeding $1 trillion by 2050.^5^ PD is projected to strike 12 million people by 2040 worldwide.^7^ Over 60,000 cases of PD are diagnosed annually in the U.S., with the economic burden reaching nearly $52 billion every year, making effective neuroprotective treatments an urgent and unmet need.^8^

The localization of the protein deposits and the chemical nature of aggregating proteins are pathologically specific. For instance, PD is linked to the aggregation of α-synuclein (α-Syn), a small cytosolic protein that is mainly located in synaptic terminals.^9^ Although the physiological function of α-Syn remains largely unknown,^10−12^ this protein plays an important role in synaptic plasticity, the inflammatory response, and the control of neurotransmitter release in synaptic clefts.^13,14^ In solution, α-Syn is an intrinsically disordered protein that adopts α-helical structure in the presence of lipids.^15,16^ In patients with PD, α-Syn aggregates progressively form in the midbrain, hypothalamus, and thalamus.^17^ In the case of AD, the abnormal aggregation of amyloid beta (Aβ) peptide and hyperphosphorylated tau protein is thought to mediate the sequential loss of neurons in the frontal cortex of the brain.^18^ Injection amyloidosis and diabetes type 2 are linked to the aggregation of insulin, a small protein hormone that regulates the glucose level in the blood.^19^ Insulin injections in the skin derma cause a rapid increase in the concentration of the protein in a small volume of tissue, which can trigger insulin aggregation. Similar processes could take place in the pancreas upon overproduction of insulin, which is observed in patients with diabetes type 2. Finally, hereditary systemic amyloidosis (HSA) is caused by an abrupt aggregation of lysozyme, a major player in the innate immune system.^20^ This extracellular protein can aggregate, forming massive deposits in the livers and kidneys of individuals diagnosed with HSA.

## Amyloidogenic Diseases on the Molecular Level

Although the exact cause of amyloidogenic pathologies is unknown, protein misfolding and a high local concentration of proteins are the major factors that trigger protein aggregation. Computational analysis of protein sequences performed by the Eisenberg group revealed that the presence of certain amino acid motifs is the key determinant of the proteins’ self-assembly. These amino acid sequences form a β-sheet (also known as a “dry zipper”) structure that is stabilized by hydrogen bonding.^21^ Thus, the energy minimization of misfolded proteins with such amino acid sequences drives their association into the β-sheet structure. As a result, protein oligomers are formed. These transient species exhibit a large diversity of shapes and forms. Amyloid oligomers can template the aggregation of misfolded proteins, which results in the formation of fibrils. In this case, two β-sheets associate together plane-to-plane, forming an even more thermodynamically stable cross β-sheet, which stretches micrometers in length in the direction perpendicular to the peptide strands in β-sheets. Electrostatic energy minimization makes fibrils twist and coil, forming large supramolecular assemblies with both twisted and tape-like morphologies.

## Structural Organization of Amyloid Oligomers and Fibrils

Utilization of the solid-state nuclear magnetic resonance (ss-NMR) and cryo-electron microscopy (cryo-EM) allowed for the elucidation of the secondary structure of amyloid fibrils with angstrom spatial resolution.^22,23^ It was found that fibrils formed by different amyloidogenic proteins share very similar structures. Specifically, fibrils are composed of two β-sheets with ∼4.7 Å interstrand distances spaced ∼10 Å between each other. Some fibrils may have three β-sheets arranged in a triangle. These variabilities in the arrangement of β-sheets in one fibril are the underlying cause of fibril polymorphism.

The transient nature and morphological heterogeneity of amyloid oligomers limit the use of ss-NMR and cryo-EM for their structural characterization. Our group demonstrated that this problem could be overcome with nano-infrared spectroscopy, also known as atomic force microscopy infrared (AFM-IR) spectroscopy.^24^ In AFM-IR, a metallized scanning probe is first used to reveal the topology of the aggregate.^25^ Next, the probe can be positioned above the aggregate of interest.^26^ Pulsed IR light is then sent to the sample to cause thermal expansion of the analyzed aggregates. Such thermal expansion is recorded by the scanning probe and converted to the IR spectra that can be used to reveal the secondary structure of individual amyloid oligomers.^27,28^ Furthermore, by using AFM-IR, chemical maps of the protein aggregates can be obtained. Such maps are combinations of the AFM maps of the specimens and their IR signatures.

Elucidation of the secondary structure is achieved by the fitting of the amide I band, which primarily originates from the C=O vibration of the peptide bond.^29^ Localization of the amide band at ∼1630 and 1695 cm^–1^ indicates the predominance of parallel and antiparallel β-sheet secondary structure, respectively. At the same time, the shift of amide I to ∼1645 and 1660 cm^–1^ is indicative of α-helix and unordered protein secondary structure, respectively.^2,3,30−38^

**Figure 1:**
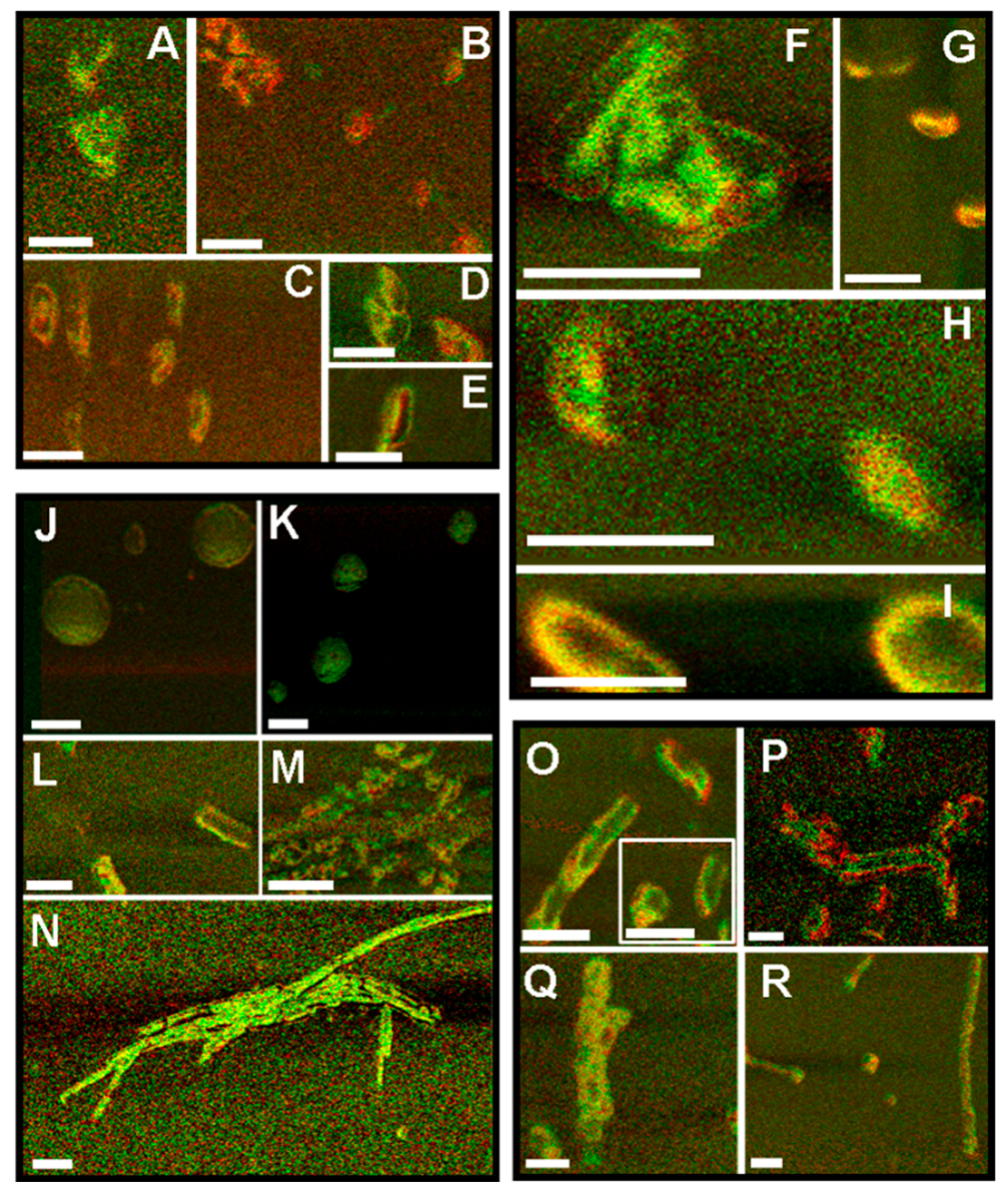
Overlapped infrared and AFM chemical maps at 1655 and 1624 cm^–1^ for D1 (A–E), D2 (F–I), D3 (J–N), and D7 (O–R) samples. Random coil and α-helical conformations (1655 cm^–1^) and the β-sheet (1624 cm^–1^) are marked by pseudocolor red and green, respectively. Scale bars: A–I and O–R, 100 nm; J–N, 250 nm. Reproduced from ref (24). Copyright 2020 American Chemical Society.

Using AFM-IR, Zhou and Kurouski investigated changes in the secondary structure of α-Syn aggregates formed at different stages of protein aggregation.^24^ It was found that at the early time points, drastically different from the perspective of their secondary structure, oligomers were found (Figure 1). Some of them were dominated by parallel β-sheets, whereas others have a mixture of α-helix and unordered protein secondary structures. Zhou and Kurouski also found that, on average, early stage oligomers possessed a high amount of antiparallel β-sheet secondary structure that progressively decreased as the oligomers developed into fibrillar species and then mature fibrils, which had parallel β-sheet secondary structure. It was suggested that α-Syn aggregation is driven by the conversion of antiparallel to parallel β-sheet secondary structure.

**Figure 2:**
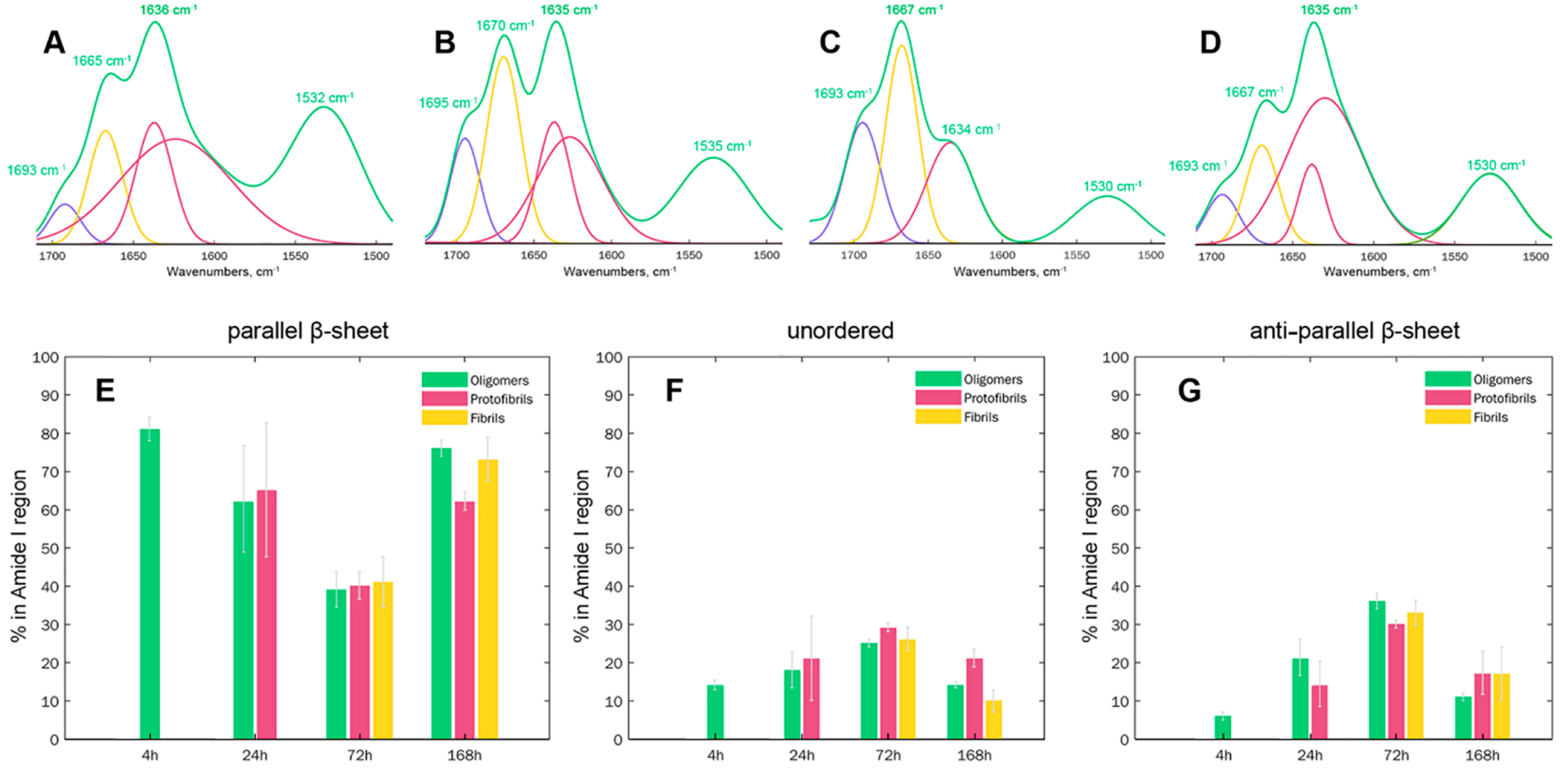
Structural analysis of Aβ_1–42_ aggregates formed at different states of protein aggregation. Averaged AFM-IR spectra (green) acquired from individual aggregates observed 4 h (A), 24 h (B), 72 h (C), and 168 h (D) after the initiation of Aβ_1–42_ aggregation reveals the presence of an antiparallel β-sheet (1693–1695 cm^–1^), unordered protein (1665–1670 cm^–1^), and a parallel β-sheet (1634–1636 cm^–1^) in their structure. Spectral fitting of the amide I region (1693–1634 cm^–1^) enabled the quantification of relative contributions of the antiparallel β-sheet (purple), unordered protein (yellow), and parallel β-sheet (red and maroon) protein secondary structures in oligomers, proto-fibrils, and fibrils (E–G). Reproduced from ref (35). Copyright 2022 American Chemical Society.

Expanding upon this, Zhaliazka and Kurouski investigated structural changes that took place upon aggregation of the amyloid β_1–42_ peptide (Aβ_1–42_).^35^ Using AFM-IR, the researchers found that Aβ_1–42_ first formed oligomers with parallel β-sheet structures that had much slower rates of propagation into fibrils. Right after their appearance, the researchers detected oligomers with an antiparallel β-sheet, which rapidly propagated into protofilaments and fibrils (Figure 2). By 72 h after the initiation of Aβ_1–42_ aggregation, nearly equal amounts of aggregates with parallel and antiparallel β-sheet secondary structures were observed. Finally, Aβ_1–42_ aggregates with antiparallel β-sheets remained as a subpopulation in the late stages of protein aggregation, and oligomers and fibrils with parallel β-sheets were dominated.

### Role of Lipids in Amyloidosis

Microscopic examination of Lewy bodies, protein deposits found in PD brains, revealed the presence of fragments of lipid-rich membranes, organelles, and vesicles.^39,40^ Similar observations were made for amyloid plaques that are formed in the brain frontal cortex upon AD. Furthermore, many amyloidogenic proteins, such as α-Syn and Aβ_1–42_, facilitate or perform physiologically important functions in the plasma membranes. These and other pieces of experimental evidence suggested that lipid bilayers can play an important role in the onset and progression of amyloidogenic diseases.^36,41^

### Lipids Uniquely Alter the Rate of Protein Aggregation

Experimental findings reported by Galvagnion demonstrated that the α-Syn aggregation rate could be altered by lipids.^13,42,43^ Specifically, it was found that the rate of α-Syn aggregation in the presence of large unilamellar vesicles (LUVs) of dimyristoylphosphatidylserine (DMPS) was substantially greater compared to the rate of α-Syn aggregation in a lipid-free environment. Furthermore, Galvagnion and co-workers demonstrated that the rates of α-Syn aggregation depended on the lipid-to-protein ratio (L:P ratio). It was proposed that LUVs at low concentrations serve as the nucleation sites for α-Syn, which results in the acceleration of protein aggregation. At the same time, an increase in the surface area of lipid bilayers lowers the probability of protein–protein interactions, which are critical for α-Syn aggregation. Therefore, with an increase in the L:P ratio, a subsequent decrease in the rate of α-Syn aggregation was observed. A growing body of evidence suggests that other phospho- and sphingolipids exerted similar effects on the aggregation of α-Syn.^2,38^ Specifically, it was found that LUVs of phosphatidylcholine (PC) and phosphatidic acid (PA) were able to accelerate the rate of α-Syn aggregation compared to the rate of fibril formation in the lipid-free environment.^2,38^

Zhaliazka and co-workers recently reported that phospholipids and cholesterol could drastically accelerate the rate of Aβ_1–42_ aggregation.^36^ Specifically, it was found that LUVs of both PC and cardiolipin (CL) could significantly increase the aggregation rate of Aβ_1–42_. Furthermore, the researchers reported that cholesterol, if present in a 5% ratio in the PC LUVs, did not alter the rate of oligomer formation. However, it accelerated the elongation of such oligomers into fibrils, which was not evident for PC alone.

**Figure 3:**
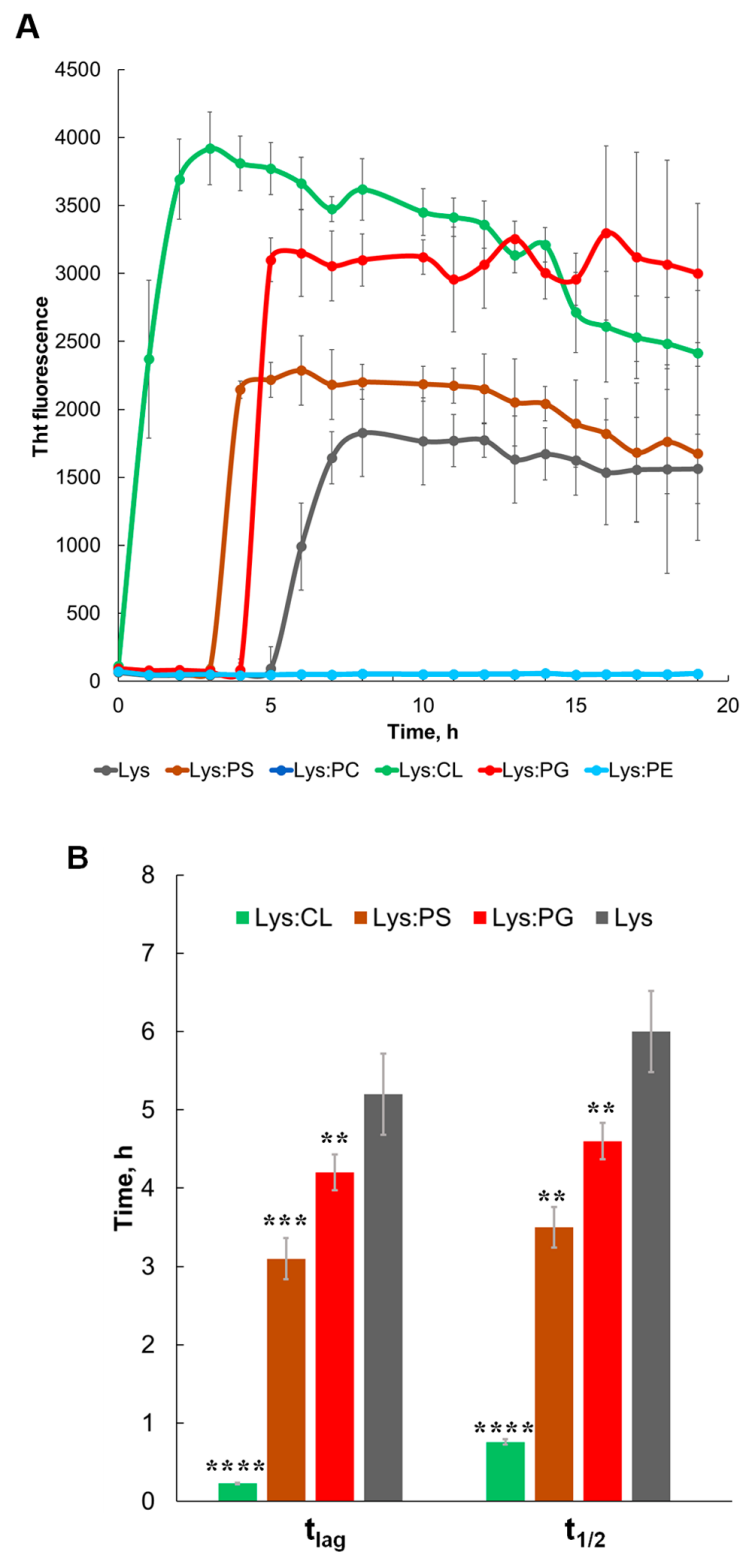
Negatively charged phospholipids drastically accelerate whereas zwitterionic phospholipids strongly inhibit lysozyme aggregation. (A) ThT aggregation kinetics with (B) corresponding values of *t*_lag_ and *t*_1/2_ of lysozyme in the lipid-free environment (gray) as well as in the presence of PG (red), PS (brown), CL (green), PC (blue), and PE (light blue). Each kinetic curve is the average of three independent measurements. Reproduced from ref (37). Copyright 2022 American Chemical Society.

Our group also investigated the effect of different phospho- and sphingolipids on the rate of insulin aggregation.^3,30−34^ It was found that negatively charged phospholipids, such as CL, PS, PA, and phosphatidylglycerol (PG), accelerated the rate of insulin aggregation whereas zwitterionic lipids, such as PC and phosphatidylethanolamine (PE), on the opposite, strongly inhibited fibril formation.^44^ It should be noted that ceramide (CER) was found to enhance insulin aggregation, whereas sphingomyelin (SM), on the other hand, slowed down fibril formation.^34^ Similar results were reported by our group for lysozyme.^3,37^ Specifically, the researchers found that PC and PE, similar to insulin, inhibited lysozyme aggregation, whereas negatively charged PS, PG, and CL strongly accelerated lysozyme fibril formation (Figure 3).^3^ It should be noted that from all analyzed lipids, CL, which at physiological pH possessed two negative charges, exhibited the strongest enhancement of lysozyme aggregation compared to the negatively charged (−1) PS and PG. Based on these results, we could conclude that the rate of protein aggregation directly depends on the net charge of the lipid. Zhaliazka and co-workers discovered that the enhancement rate of protein aggregation directly depends not only on the chemical structure of the lipid, but also on the P:L ratio.^45^ Specifically, PS and CL exhibited a much stronger enhancement of lysozyme aggregation if they were present at 1:10 and 1:5 compared to 1:1 P:L ratios.

Recently, Frese and co-workers found that in addition to the net charge of the lipid polar head and P:L ratios, the length and saturation of FAs could uniquely alter the aggregation rates of lysozyme.^46^ Specifically, lysozyme exhibited the shortest lag phase in the presence of equimolar concentrations of PS with 14 carbon-atom-long (14:0) FAs (1,2-dimyristoyl-*sn*-glycero-3-phospho-l-serine, DMPS) compared to PS with 18-carbon-long (18:0) FAs (1,2-distearyl-*sn*-glycero-3-phospho-l-serine, DSPS). Similar results were reported by Matveyenka and co-workers for insulin.^31^ Finally, the presence of double bonds in FAs of PS enabled a much greater acceleration of both insulin and lysozyme aggregation compared to fully saturated FAs with the same length of FAs.^31^ Specifically, 1-palmitoyl-2-oleoyl-*sn*-glycero-3-phospho-l-serine (POPS) and 1,2-dioleoyl-*sn*-glycero-3-phospho-l-serine, (DOPS), which possess one and two double bonds, respectively, exhibited a much stronger enhancement of lysozyme aggregation compared to fully saturated DSPS. Similar results were reported by Matveyenka and co-workers for saturated and unsaturated CL.^30^ However, Matveyenka and co-workers found that unsaturation did not alter the inhibition properties of PC on the insulin aggregation.^30^ Finally, our group discovered that PA with 16-carbon-long FAs (C16:0) was found to inhibit insulin aggregation, whereas PA with 18-carbon-long FAs (C18:0), on the other hand, strongly enhanced the protein aggregation.^32^ In summary, these results revealed that a large number of factors can alter the rate of protein aggregation in the presence of lipids. In particular, it was found that (1) the net charge of the lipid; (2) P:L ratio; (3) length of FAs; (4) saturation of FAs; and (5) the chemical structure of the lipid itself play an important role in the aggregation rate of amyloidogenic proteins.

### Structure and Morphology of Amyloid Aggregates Formed in the Presence of Lipids

Experimental results reported by our group and other research groups demonstrated that lipids uniquely altered the morphology and secondary structure of amyloid aggregates formed in their presence.^2,3,30−38,44,45^ Specifically, both insulin and lysozyme formed spherical oligomers in the presence of PC and PE.^37,44^ These aggregates dominated by unordered protein secondary structure possessing very few if any β-sheets. At the same time, insulin and lysozyme formed fibrils in the presence of negatively charged lipids, such as CL, PS, and PG.^37,44^ These aggregates were dominated by a parallel β-sheet secondary structure.

**Figure 4:**
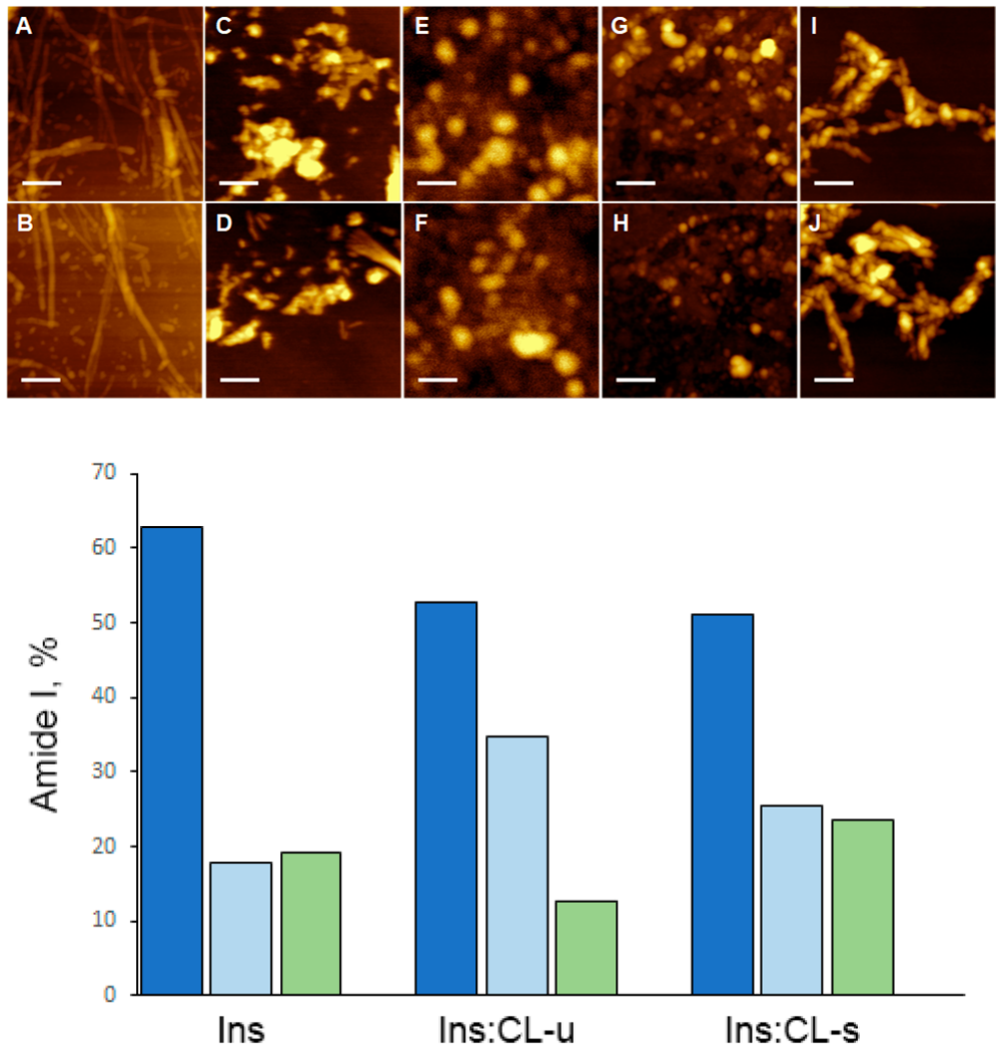
Lipids uniquely alter the morphologies of insulin aggregates. AFM images (top) of Ins:CL-u (A and B), Ins:CL-s (C and D), Ins:PC-u (E and F), Ins:PC-s (G and H), and insulin aggregates grown in the lipid-free environment (I and J). Histograms (bottom) of relative contributions of the parallel β-sheet (blue), unordered protein secondary structure (light blue), and antiparallel β-sheet (green) in amide I of AFM-IR spectra collected from two populations (A and B) of Ins:PC-u and Ins:PC-s (top) and Ins:CL-u and Ins:CL-s (bottom) together with insulin aggregates (Ins) grown in the lipid-free environment. Scale bars are 200 nm. Reproduced from ref (30). Copyright 2022 American Chemical Society.

AFM imaging of insulin fibrils formed in the presence of unsaturated CL revealed the presence of long fibrillar structures of 6–8 nm in height that stretched for micrometers in length (Figure 4).^30^ Such aggregates did not form in the presence of saturated CL. Instead, Matveyenka and co-workers found relatively short (∼200 nm in length) fibrils formed by insulin in the presence of saturated CL.^30^ These results demonstrated that the unsaturation of FAs in CL promoted insulin assembly into long fibrils that could not be formed in the presence of its saturated analog. It should be noted that in the lipid-free environment insulin formed fibrillar assemblies with a large variety of lengths that were, on average, 12 nm in height. Using AFM-IR, Matveyenka and co-workers also found that the secondary structure of insulin fibrils formed in the presence of saturated and unsaturated CL was different from the secondary structure of insulin fibrils formed in the lipid-free environment.^30^

It should be noted that both insulin and lysozyme aggregates formed in the presence of lipids possessed corresponding lipids in their structure.^2,3,30−38,44,45^ Similar results were reported by Zhaliazka and co-workers for Aβ_1–42_ oligomers and fibrils formed in the presence of CL, PC, and the PC:cholesterol mixture.^36^ It was found that Aβ_1–42_ aggregates exhibit a vibrational band centered at around 1730 cm^–1^, which could be assigned to the C=O vibration of lipids from the AFM-IR spectra acquired from these species. Based on these results, the researchers concluded that CL and PC were present in the structure of Aβ_1–42_ oligomers and fibrils.^36^ Dou and co-workers discovered the presence of PC and PS in α-Syn oligomers formed in the early and late stages of protein aggregation in the presence of these phospholipids.^2,38^ It should be noted that in addition to the presence of lipids such aggregates had drastically different secondary structures compared to the oligomers formed in the lipid-free environment.

**Figure 5:**
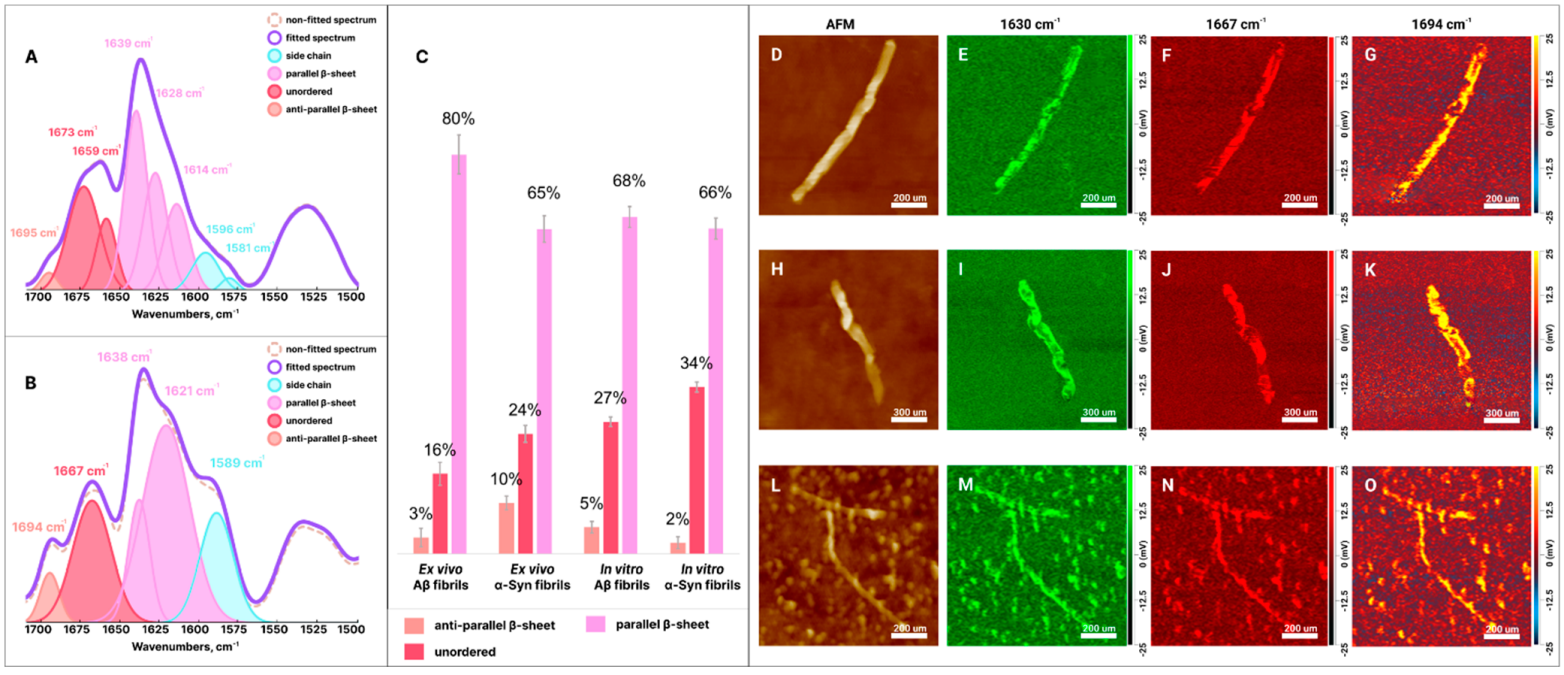
Averaged AFM-IR spectra acquired from *in vitro* Aβ (A) and α-Syn (B) fibrils. Histogram (C) of relative contributions of parallel- and antiparallel β-sheets and unordered protein in *ex vivo* Aβ and α-Syn, as well as in *in vitro* Aβ and α-Syn fibrils. AFM (D–H and L) images of *in vitro* Aβ (D–K) and α-Syn (L–O) fibrils with the corresponding nano-IR images reveal the nanoscale distribution of their parallel β-sheet (E, I, and M), unordered protein (F, J, and N), and antiparallel β-sheet (G, K, and O). Reproduced with permission from ref (1). Copyright 2023 Wiley.

These experimental findings are consistent with the recently reported results by Zhaliazka and Kurouski (Figure 5).^1^ The researchers extracted both Aβ and α-Syn fibrils from the brains of patients with AD and PD, respectively. It was found that α-Syn fibrils possessed lipids in their structure, whereas no lipids were observed in the Aβ fibrils. It was also demonstrated that *ex vivo*-extracted Aβ and α-Syn fibrils had drastically different secondary structure compared to those formed *in vitro* (Figure 5).^1^

### Toxicity of Amyloid Aggregates Formed in the Presence of Lipids

**Figure 6:**
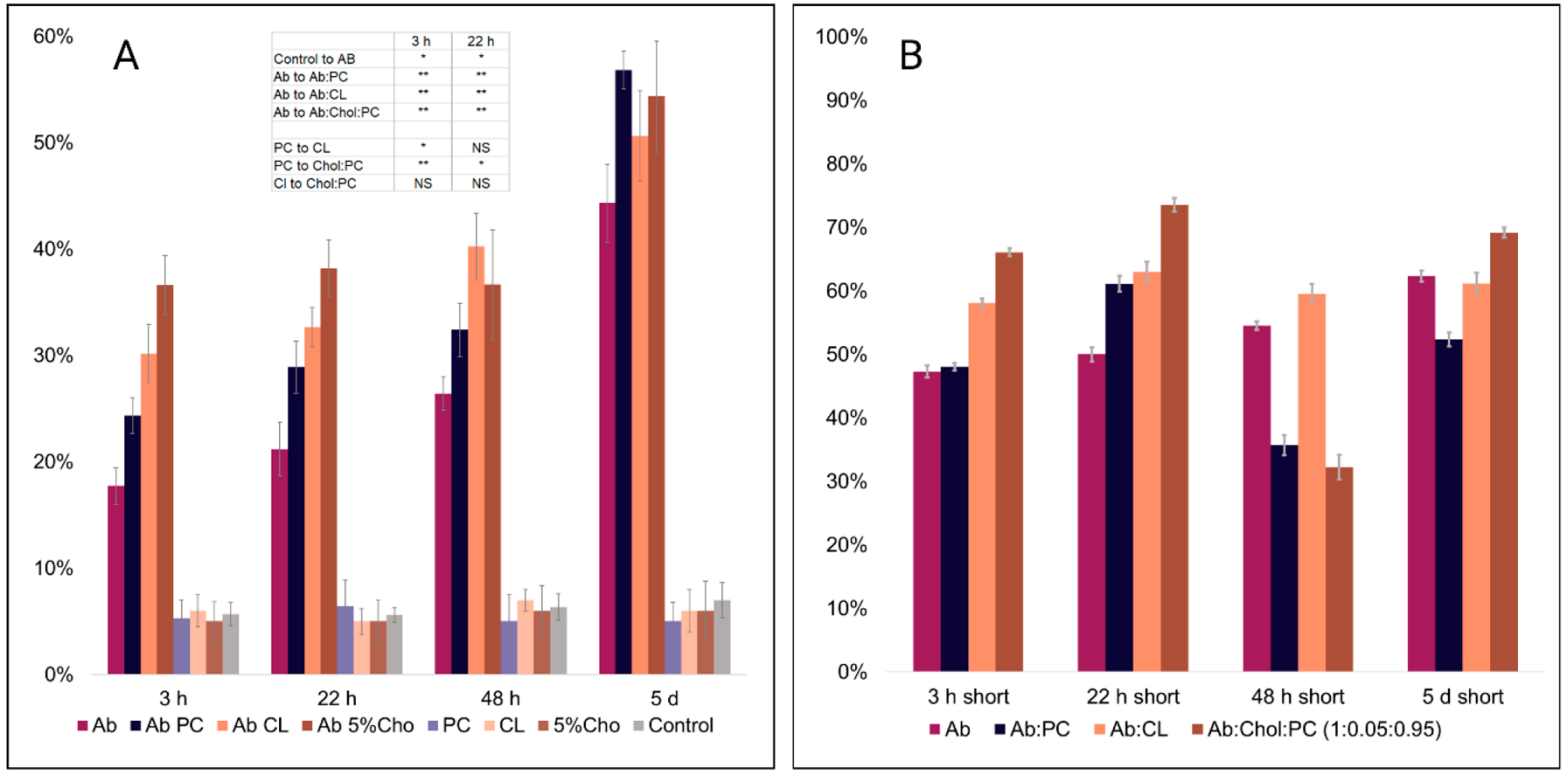
(A) Results of LDH toxicity assay showing the relative toxicity of Aβ aggregates formed at different stages of protein aggregation. (B) Content of parallel β-sheet (1610–1640 cm^–1^) in an average of short Aβ_1–42_ oligomers by all-time points. Reproduced with permission from ref (34). Copyright 2023 Elsevier.

Our group discovered that lipids uniquely alter the toxicity of protein aggregates that are formed in their presence. For instance, phospho- and sphingolipids with saturated FAs lowered the toxicity of both insulin and lysozyme fibrils (Figure 6).^3,33,34,44,46^ However, this effect was not observed for the phospholipids with unsaturated FAs.^30^

Zhaliazka and co-workers found that Aβ_1–42_ fibrils formed in the presence of lipids exerted much greater levels of cell toxicity compared to the aggregates formed in the lipid-free environment.^36^ Aβ_1–42_ fibrils formed in the presence of the PC:cholesterol mixture were found to be far more toxic than Aβ_1–42_:PC and Aβ_1–42_:CL fibrils (Figure 6). Furthermore, it was found that the cytotoxicity of Aβ_1–42_ fibrils had a direct relationship with the amount of parallel β-sheet in these aggregates.

**Figure 7:**
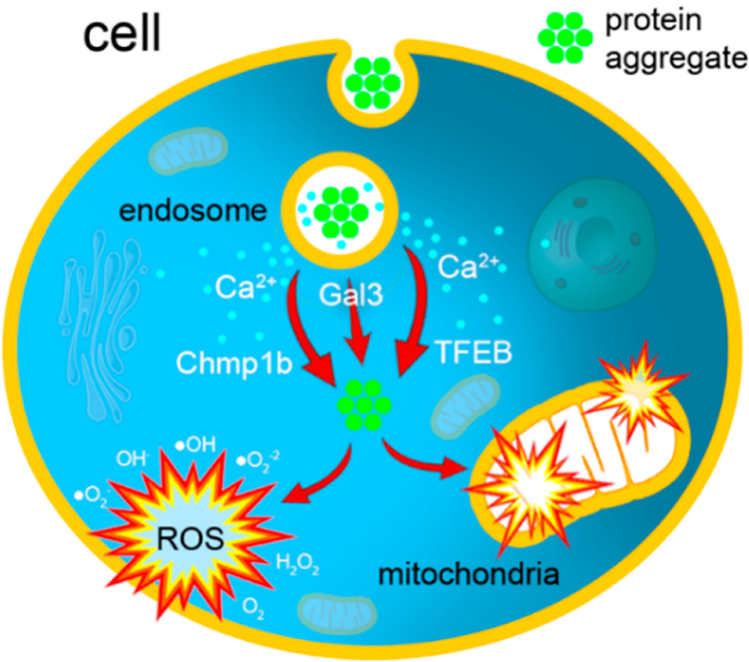
Mechanism of cell toxicity exerted by insulin aggregates.

Our group also investigated mechanisms by which amyloid aggregates exerted cell toxicity. Matveyenka and co-workers found that insulin fibrils damage cell endosomes, which triggers Ca^2+^ leakage, endosomal repair, and *de novo* biogenesis of endosomes.^34^ The researchers also found that the extent to which fibrils damage cell endosomes is strongly correlated with the secondary structure of these protein aggregates. Once they escape from the endosomes, insulin fibrils appear in the cell cytosol, where they can damage the endoplasmic reticulum and cell mitochondria (Figure 7). This ultimately causes cell death.

## Concluding Remarks

The most recent findings summarized in this Account demonstrate that lipid bilayers strongly alter the rates of protein aggregation.^3,33,34,44,46^ The extent to which lipids alter the rate of protein aggregation directly depends on the structure of the protein, the net charge of the lipid, and the length and saturation of FAs in the lipid. There are also numerous pieces of experimental evidence indicating that lipids change the secondary structure and morphology of amyloid oligomers and fibrils formed in their presence. Such fibrils exert drastically different levels of cell toxicity compared to amyloid oligomers and fibrils formed in the lipid-free environments.^3,33,34,44,46^

It should be noted that other factors, such as the size of lipid vesicles and their phase transition, can strongly affect the rate of protein aggregation. Specifically, Terakawa and co-workers found that small unilamellar vesicles (SUVs) enable much faster aggregation of amyloid β1-40 peptide compared to LUVs that had the same composition of lipids.^47^ This observation can be explained by a higher number of defects of lipids in SUVs than in LUVs. Consequently, lipids in such membrane defects become more accessible to amyloidogenic proteins that adsorb onto the surfaces of such vesicles. These findings highlight the important roles of membrane curvature and membrane fluidity in the aggregation properties of amyloidogenic proteins. Although the role of some of these physical parameters of lipid membranes is well understood, detailed elucidation of other factors, including the phase transition and the role of cholesterol, should be given in greater detail in the coming years.^48^

Our group recently demonstrated that not only lipids but also polyunsaturated FAs strongly accelerated the aggregation of both insulin and α-Syn.^49^ Furthermore, insulin and α-Syn fibrils formed in the presence of polyunsaturated FAs exerted greater toxicity compared to the fibrils formed in the absence of FAs.^49^ These findings show that protein:lipid and protein:FA interactions should be strongly considered upon the search for effective drug candidates that would inhibit protein aggregation. Using docking simulations, Holman and co-workers recently demonstrated that such hydrophobic forces primarily determined the interactions between insulin and FAs.^50^ As a result, inulin:FAs complexes formed fibrils with opposite supramolecular chirality compared to those developed by insulin in a lipid-free environment. It was also found that hydrophobic interactions between insulin and FAs were strongly altered by the number of carbon atoms in FAs and the degree of their unsaturation.^50^

The results summarized in this Account also suggest that the onset and progression of amyloid diseases could be linked to the pathological changes in plasma membranes. For instance, an increase in the concentration of PS in the external membrane, which takes place in the case of cell malfunctioning, can trigger the aggregation of amyloidogenic proteins.^51^ One can expect that such changes could be linked to nutrition or molecular mechanisms responsible for the maintenance of the lipid balance in the plasma and organelle membranes. Finally, the author wants to highlight that the results summarized in this Account were obtained in the *in vitro* experiments and therefore require additional validation in living systems to fully understand the effect of lipids on amyloidosis.

## References

[ref1] Zhaliazka K.; Kurouski D. Nanoscale Imaging of Individual Amyloid Aggregates Extracted from Brains of Alzheimer and Parkinson Patients Reveals Presence of Lipids in Alpha-Synuclein but Not in Amyloid Beta(1–42) Fibrils. Protein Sci. 2023, 32, e4598 10.1002/pro.4598. 36823759 PMC10019452

[ref2] Dou T.; Zhou L.; Kurouski D. Unravelling the Structural Organization of Individual Alpha-Synuclein Oligomers Grown in the Presence of Phospholipids. J. Phys. Chem. Lett. 2021, 12, 4407–4414. 10.1021/acs.jpclett.1c00820. 33945282

[ref3] Matveyenka M.; Zhaliazka K.; Rizevsky S.; Kurouski D. Lipids Uniquely Alter Secondary Structure and Toxicity of Lysozyme Aggregates. FASEB J. 2022, 36, e22543 10.1096/fj.202200841R. 36094052 PMC10427241

[ref4] Rizevsky S.; Matveyenka M.; Kurouski D. Nanoscale Structural Analysis of a Lipid-Driven Aggregation of Insulin. J. Phys. Chem. Lett. 2022, 13, 2467–2473. 10.1021/acs.jpclett.1c04012. 35266717 PMC9169669

[ref5] Gustavsson A.; Norton N.; Fast T.; Frölich L.; Georges J.; Holzapfel D.; Kirabali T.; Krolak-Salmon P.; Rossini P. M.; Ferretti M. T.; Lanman L.; Chadha A. S.; van der Flier V. M. Global estimates on the number of persons across the Alzheimer’s disease continuum. Alzh. Diment. 2023, 19, 658–670. 10.1002/alz.12694. 35652476

[ref6] Alzheimer’s Disease Facts and Figures, Alzheimers Dement 2022, https://www.alz.org/alzheimers-dementia/facts-figures.

[ref7] Chen J. Parkinson’s Disease: Health-Related Quality of Life, Economic Cost, and Implications of Early Treatment. Am. J. Manag. Care 2010, 16, S87–S93. 20297871

[ref8] Bengoa-Vergniory N.; Roberts R. F.; Wade-Martins R.; Alegre-Abarrategui J. Alpha-Synuclein Oligomers: A New Hope. Acta Neuropathol. 2017, 134, 819–838. 10.1007/s00401-017-1755-1. 28803412 PMC5663814

[ref9] Alecu I.; Bennett S. A. L. Dysregulated Lipid Metabolism and Its Role in Alpha-Synucleinopathy in Parkinson’s Disease. Front. Neurosci. 2019, 13, 328 10.3389/fnins.2019.00328. 31031582 PMC6470291

[ref10] Walsh D. M.; Klyubin I.; Fadeeva J. V.; Cullen W. K.; Anwyl R.; Wolfe M. S.; Rowan M. J.; Selkoe D. J. Naturally Secreted Oligomers of Amyloid Beta Protein Potently Inhibit Hippocampal Long-Term Potentiation in Vivo. Nature 2002, 416, 535–9. 10.1038/416535a. 11932745

[ref11] Vilar M.; Chou H. T.; Luhrs T.; Maji S. K.; Riek-Loher D.; Verel R.; Manning G.; Stahlberg H.; Riek R. The Fold of Alpha-Synuclein Fibrils. Proc. Nat. Acad. Sci. U. S. A. 2008, 105, 8637–42. 10.1073/pnas.0712179105. PMC2438424 18550842

[ref12] Heise H.; Hoyer W.; Becker S.; Andronesi O. C.; Riedel D.; Baldus M. Molecular-Level Secondary Structure, Polymorphism, and Dynamics of Full-Length Alpha-Synuclein Fibrils Studied by Solid-State Nmr. Proc. Nat. Acad. Sci. U. S. A. 2005, 102, 15871–6. 10.1073/pnas.0506109102. PMC1276071 16247008

[ref13] Galvagnion C. The Role of Lipids Interacting with -Synuclein in the Pathogenesis of Parkinson’s Disease. J. Parkins. Dis. 2017, 7, 433–450. 10.3233/JPD-171103. 28671142

[ref14] Kurouski D.; Luo H.; Sereda V.; Robb F. T.; Lednev I. K. Deconstruction of Stable Cross-Beta Fibrillar Structures into Toxic and Nontoxic Products Using a Mutated Archaeal Chaperonin. ACS Chem. Biol. 2013, 8, 2095–101. 10.1021/cb400238a. 23875676

[ref15] Galvagnion C.; Buell A. K.; Meisl G.; Michaels T. C.; Vendruscolo M.; Knowles T. P.; Dobson C. M. Lipid Vesicles Trigger Alpha-Synuclein Aggregation by Stimulating Primary Nucleation. Nat. Chem. Biol. 2015, 11, 229–34. 10.1038/nchembio.1750. 25643172 PMC5019199

[ref16] Singh Y.; Sharpe P. C.; Hoang H. N.; Lucke A. J.; McDowall A. W.; Bottomley S. P.; Fairlie D. P. Amyloid Formation from an Alpha-Helix Peptide Bundle Is Seeded by 3(10)-Helix Aggregates. Chemistry 2011, 17, 151–60. 10.1002/chem.201002500. 21207612

[ref17] Surmeier D. J.; Obeso J. A.; Halliday G. M. Selective Neuronal Vulnerability in Parkinson Disease. Nat. Rev. Neurosci. 2017, 18, 101–113. 10.1038/nrn.2016.178. 28104909 PMC5564322

[ref18] Gouras G. K.; Tampellini D.; Takahashi R. H.; Capetillo-Zarate E. Intraneuronal Beta-Amyloid Accumulation and Synapse Pathology in Alzheimer’s Disease. Acta Neuropathol. 2010, 119, 523–41. 10.1007/s00401-010-0679-9. 20354705 PMC3183823

[ref19] Shikama Y.; Kitazawa J.; Yagihashi N.; Uehara O.; Murata Y.; Yajima N.; Wada R.; Yagihashi S. Localized Amyloidosis at the Site of Repeated Insulin Injection in a Diabetic Patient. Int. Med. 2010, 49, 397–401. 10.2169/internalmedicine.49.2633. 20190472

[ref20] Pleyer C.; Flesche J.; Saeed F. Lysozyme Amyloidosis - a Case Report and Review of the Literature. Clin. Nephrol. Case Stud. 2015, 3, 42–45. 10.5414/CNCS108538. 29043133 PMC5437999

[ref21] Sawaya M.; Sambashivan S.; Nelson R.; Ivanova M. I.; Sievers S. A.; Apostol M. I.; Thompson M. J.; Balbirnie M.; Wiltzius J. J.W.; McFarlane H. T.; Madsen A. Ø.; Riekel C.; Eisenberg D. Atomic Structures of Amyloid Cross-Beta Spines Reveal Varied Steric Zippers. Nature 2007, 447, 453–457. 10.1038/nature05695. 17468747

[ref22] Tycko R. Solid-State Nmr Studies of Amyloid Fibril Structure. Annu. Rev. Phys. Chem. 2011, 62, 279–99. 10.1146/annurev-physchem-032210-103539. 21219138 PMC3191906

[ref23] Guerrero-Ferreira R.; Taylor N. M.; Mona D.; Ringler P.; Lauer M. E.; Riek R.; Britschgi M.; Stahlberg H. Cryo-Em Structure of Alpha-Synuclein Fibrils. Elife 2018, 7, 10.7554/eLife.36402. PMC6092118 29969391

[ref24] Zhou L.; Kurouski D. Structural Characterization of Individual Alpha-Synuclein Oligomers Formed at Different Stages of Protein Aggregation by Atomic Force Microscopy-Infrared Spectroscopy. Anal. Chem. 2020, 92, 6806–6810. 10.1021/acs.analchem.0c00593. 32347706

[ref25] Kurouski D.; Dazzi A.; Zenobi R.; Centrone A. Infrared and Raman Chemical Imaging and Spectroscopy at the Nanoscale. Chem. Soc. Rev. 2020, 49, 3315–3347. 10.1039/C8CS00916C. 32424384 PMC7675782

[ref26] Dou T.; Li Z.; Zhang J.; Evilevitch A.; Kurouski D. Nanoscale Structural Characterization of Individual Viral Particles Using Atomic Force Microscopy Infrared Spectroscopy (Afm-Ir) and Tip-Enhanced Raman Spectroscopy (Ters). Anal. Chem. 2020, 92, 11297–11304. 10.1021/acs.analchem.0c01971. 32683857

[ref27] Centrone A. Infrared Imaging and Spectroscopy Beyond the Diffraction Limit. Annu. Rev. Anal. Chem. 2015, 8, 101–126. 10.1146/annurev-anchem-071114-040435. 26001952

[ref28] Ruggeri F. S.; Flagmeier P.; Kumita J. R.; Meisl G.; Chirgadze D. Y.; Bongiovanni M. N.; Knowles T. P. J.; Dobson C. M. The Influence of Pathogenic Mutations in Alpha-Synuclein on Biophysical and Structural Characteristics of Amyloid Fibrils. ACS Nano 2020, 14, 5213–5222. 10.1021/acsnano.9b09676. 32159944

[ref29] Kurouski D.; Van Duyne R. P.; Lednev I. K. Exploring the Structure and Formation Mechanism of Amyloid Fibrils by Raman Spectroscopy: A Review. Analyst 2015, 140, 4967–80. 10.1039/C5AN00342C. 26042229

[ref30] Matveyenka M.; Rizevsky S.; Kurouski D. Unsaturation in the Fatty Acids of Phospholipids Drastically Alters the Structure and Toxicity of Insulin Aggregates Grown in Their Presence. J. Phys. Chem. Lett. 2022, 13, 4563–4569. 10.1021/acs.jpclett.2c00559. 35580189 PMC9170185

[ref31] Matveyenka M.; Rizevsky S.; Kurouski D. The Degree of Unsaturation of Fatty Acids in Phosphatidylserine Alters the Rate of Insulin Aggregation and the Structure and Toxicity of Amyloid Aggregates. FEBS Lett. 2022, 596, 1424–1433. 10.1002/1873-3468.14369. 35510803 PMC9197964

[ref32] Matveyenka M.; Rizevsky S.; Kurouski D. Length and Unsaturation of Fatty Acids of Phosphatidic Acid Determines the Aggregation Rate of Insulin and Modifies the Structure and Toxicity of Insulin Aggregates. ACS Chem. Neurosci. 2022, 13, 2483–2489. 10.1021/acschemneuro.2c00330. 35930674

[ref33] Matveyenka M.; Rizevsky S.; Kurouski D. Amyloid Aggregates Exert Cell Toxicity Causing Irreversible Damages in the Endoplasmic Reticulum. Biochim. Biophys. Acta Mol. Basis Dis. 2022, 1868, 166485 10.1016/j.bbadis.2022.166485. 35840040 PMC10424722

[ref34] Matveyenka M.; Rizevsky S.; Pellois J. P.; Kurouski D. Lipids Uniquely Alter Rates of Insulin Aggregation and Lower Toxicity of Amyloid Aggregates. Biochim. Biophys. Acta Mol. Cell. Biol. Lipids 2023, 1868, 159247 10.1016/j.bbalip.2022.159247. 36272517 PMC10401553

[ref35] Zhaliazka K.; Kurouski D. Nanoscale Characterization of Parallel and Antiparallel Beta-Sheet Amyloid Beta 1–42 Aggregates. ACS Chem. Neurosci. 2022, 13, 2813–2820. 10.1021/acschemneuro.2c00180. 36122250 PMC10405294

[ref36] Zhaliazka K.; Kurouski D. Lipids Uniquely Alter the Secondary Structure and Toxicity of Amyloid Beta 1–42 Aggregates. FEBS J. 2023, 290, 3203–3220. 10.1111/febs.16738. 36705524 PMC10389563

[ref37] Zhaliazka K.; Rizevsky S.; Matveyenka M.; Serada V.; Kurouski D. Charge of Phospholipids Determines the Rate of Lysozyme Aggregation but Not the Structure and Toxicity of Amyloid Aggregates. J. Phys. Chem. Lett. 2022, 13, 8833–8839. 10.1021/acs.jpclett.2c02126. 36111888 PMC10405293

[ref38] Dou T.; Kurouski D. Phosphatidylcholine and Phosphatidylserine Uniquely Modify the Secondary Structure of Alpha-Synuclein Oligomers Formed in Their Presence at the Early Stages of Protein Aggregation. ACS Chem. Neurosci. 2022, 13, 2380–2385. 10.1021/acschemneuro.2c00355. 35904551 PMC10405296

[ref39] Shahmoradian S. H.; Lewis A. J.; Genoud C.; Hench J.; Moors T. E.; Navarro P. P.; Castaño-Díez D.; Schweighauser G.; Graff-Meyer A.; Goldie K. N.; Sütterlin R.; Huisman E.; Ingrassia A.; de Gier Y.; Rozemuller A. J. M.; Wang J.; De Paepe A.; Erny J.; Staempfli A.; Hoernschemeyer J.; Großerüschkamp F.; Niedieker D.; El-Mashtoly S. F.; Quadri M.; Van IJcken W. F. J.; Bonifati V.; Gerwert K.; Bohrmann B.; Frank S.; Britschgi M.; Stahlberg H.; Van de Berg W. D. J.; Lauer M. E. Lewy Pathology in Parkinson’s Disease Consists of Crowded Organelles and Lipid Membranes. Nat. Neurosci. 2019, 22, 1099–1109. 10.1038/s41593-019-0423-2. 31235907

[ref40] Killinger B. A.; Melki R.; Brundin P.; Kordower J. H. Endogenous Alpha-Synuclein Monomers, Oligomers and Resulting Pathology: Let’s Talk About the Lipids in the Room. NPJ. Parkinsons Dis. 2019, 5, 23 10.1038/s41531-019-0095-3. 31728405 PMC6851126

[ref41] Rajput S.; Sani M. A.; Keizer D. W.; Separovic F. Utilizing Magnetic Resonance Techniques to Study Membrane Interactions of Amyloid Peptides. Biochem. Soc. Trans. 2021, 49, 1457–1465. 10.1042/BST20201244. 34156433 PMC8286822

[ref42] Alza N. P.; Iglesias Gonzalez P. A.; Conde M. A.; Uranga R. M.; Salvador G. A. Lipids at the Crossroad of Alpha-Synuclein Function and Dysfunction: Biological and Pathological Implications. Front. Cell. Neurosci. 2019, 13, 175 10.3389/fncel.2019.00175. 31118888 PMC6504812

[ref43] Galvagnion C.; Brown J. W.; Ouberai M. M.; Flagmeier P.; Vendruscolo M.; Buell A. K.; Sparr E.; Dobson C. M. Chemical Properties of Lipids Strongly Affect the Kinetics of the Membrane-Induced Aggregation of Alpha-Synuclein. Proc. Nat. Acad. Sci. U. S. A. 2016, 113, 7065–70. 10.1073/pnas.1601899113. PMC4932957 27298346

[ref44] Matveyenka M.; Rizevsky S.; Kurouski D. Elucidation of the Effect of Phospholipid Charge on the Rate of Insulin Aggregation and Structure and Toxicity of Amyloid Fibrils. ACS Omega 2023, 8, 12379–12386. 10.1021/acsomega.3c00159. 37033844 PMC10077570

[ref45] Zhaliazka K.; Serada V.; Matveyenka M.; Rizevsky S.; Kurouski D. Protein-to-Lipid Ratio Uniquely Changes the Rate of Lysozyme Aggregation but Does Not Significantly Alter Toxicity of Mature Protein Aggregates. Biochim. Biophys. Acta Mol. Cell. Biol. Lipids 2023, 1868, 159305 10.1016/j.bbalip.2023.159305. 36907244 PMC10405292

[ref46] Frese A.; Goode C.; Zhaliazka K.; Holman A.; Dou T.; Kurouski D. Length and Saturation of Fatty Acids in Phosphatidylserine Determine the Rate of Lysozyme Aggregation Simultaneously Altering the Structure and Toxicity of Amyloid Oligomers and Fibrils. Protein Sci. 2023, 32, e4717 10.1002/pro.4717. 37402649 PMC10364468

[ref47] Terakawa M. S.; Yagi H.; Adachi M.; Lee Y. H.; Goto Y. Small Liposomes Accelerate the Fibrillation of Amyloid Beta (1–40). J. Biol. Chem. 2015, 290, 815–26. 10.1074/jbc.M114.592527. 25406316 PMC4294504

[ref48] Jakubec M.; Barias E.; Furse S.; Govasli M. L.; George V.; Turcu D.; Iashchishyn I. A.; Morozova-Roche L. A.; Halskau O. Cholesterol-Containing Lipid Nanodiscs Promote an Alpha-Synuclein Binding Mode That Accelerates Oligomerization. FEBS J. 2021, 288, 1887–1905. 10.1111/febs.15551. 32892498

[ref49] Matveyenka M.; Zhaliazka K.; Kurouski D. Unsaturated Fatty Acids Uniquely Alter Aggregation Rate of Α-Synuclein and Insulin and Modify Secondary Structure and Toxicity of Amyloid Aggregates Formed in Their Presence. FASEB J. 2023, 37, e22972 10.1096/fj.202300003R. 37302013 PMC10405295

[ref50] Holman A. P.; Quinn K.; Kumar R.; Kmiecik S.; Ali A.; Kurouski D. Fatty Acids Reverse the Supramolecular Chirality of Insulin Fibrils. J. Phys. Chem. Lett. 2023, 14, 6935–6939. 10.1021/acs.jpclett.3c01527. 37498215 PMC10863027

[ref51] Matveyenka M.; Zhaliazka K.; Kurouski D. Concentration of Phosphatidylserine Influence Rates of Insulin Aggregation and Toxicity of Amyloid Aggregates in Vitro. ACS Chem. Neurosci. 2023, 14, 2396–2404. 10.1021/acschemneuro.3c00277. 37279439 PMC10401552

